# Supplementary material for: Association between Coronary Artery Plaque Progression and Liver Fibrosis Biomarkers in Population with Low Calcium Scores
Source: Nutrients. 2022 Jul 30;14(15):3163. doi: 10.3390/nu14153163 (PMC9370134; doi:10.3390/nu14153163)
Supplement: Supplementary file 1 [file nutrients-14-03163-s001.zip › nutrients-1833915-supplementary.pdf]

Supplement Figure S1. Interaction between DBP and the progression of atherosclerotic plaques in different subgroups.

Subgroup	Hazzard ratio (95% CI)	p for interaction
**Overall**	1.06(1.02-1.09)	
**Age**		
≧ 65 years	1.06(1.00-1.14)	0.792
< 65 years	1.05(1.01-1.10)	
**Sex**		
Male	1.04(1.00-1.08)	0.214
Female	1.11(1.00-1.23)	
**HTN**		
Yes	1.08(1.01-1.15)	0.631
No	1.06(1.01-1.11)	
**DM**		
Yes	1.11(0.98-1.25)	0.450
No	1.05(1.01-1.10)	
**Smoking**		
Yes	1.03(0.97-1.10)	0.336
No	1.07(1.03-1.12)	
**Baseline SSS**		
>=0	1.05(1.00-1.11)	0.789
=0	1.04(1.00-1.10)	
**Baseline CAC**		
>=10	1.05(0.99-1.11)	0.858
<10	1.05(1.01-1.10)	

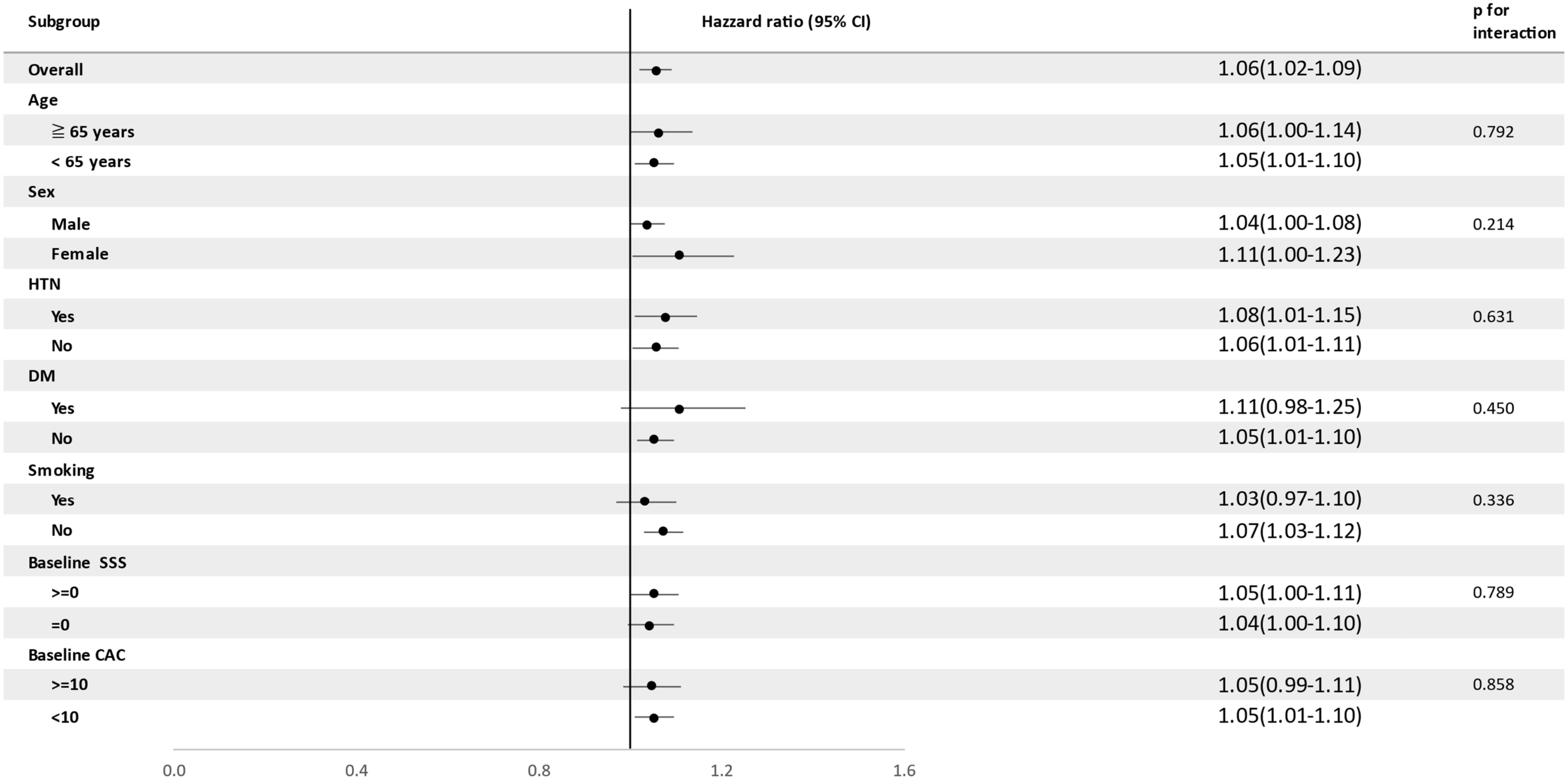

HTN=hypertension, DM=diabetes, CAC=coronary artery calcium score, SSS=segment stenosis score
